# Expression of Musashi-1 Increases in Bone Healing

**DOI:** 10.3390/ijms22073395

**Published:** 2021-03-26

**Authors:** Miguel Padial-Molina, Vicente Crespo-Lora, Clara Candido-Corral, Nati Martin-Morales, Dario Abril-Garcia, Pablo Galindo-Moreno, Pedro Hernandez-Cortes, Francisco O’Valle

**Affiliations:** 1 Department of Oral Surgery and Implant Dentistry, School of Dentistry, and Centre for Biomedical Research (CIBM), University of Granada, 18071 Granada, Spain; mipadial@ugr.es (M.P.-M.); nati@ugr.es (N.M.-M.); darioabril@ugr.es (D.A.-G.); 2 Department of Pathology, University of Granada, 18071 Granada, Spain; vcrespo1983@gmail.com (V.C.-L.); claracandidoc@gmail.com (C.C.-C.); 3 Department of Orthopedic Surgery, San Cecilio University Hospital, 18071 Granada, Spain; phc@ugr.es; 4 Department of Pathology, Institute of Biopathology and Regenerative Medicine (IBIMER, CIBM), and Institute of Biosanitary (ibs-Granada), University of Granada, 18071 Granada, Spain; fovalle@ugr.es

**Keywords:** Musashi-1, Runx2, periostin, mesenchymal stem cells, bone healing

## Abstract

Musashi-1 (MSI1) is an RNA-binding protein that regulates progenitor cells in adult and developing organisms to maintain self-renewal capacities. The role of musashi-1 in the bone healing environment and its relation with other osteogenic factors is unknown. In the current study, we analyze the expression of MSI1 in an experimental model of rat femoral bone fractures. We also analyze the relation between MSI1 expression and the expression of two osteogenic markers: periostin (POSTN) and runt-related transcription factor 2 (RUNX2). We use histological, immunohistochemical, and qPCR techniques to evaluate bone healing and the expression of MSI1, POSTN, and RUNX2 over time (4, 7, and 14 days). We compare our findings with non-fractured controls. We find that in bone calluses, the number of cells expressing MSI1 and RUNX2 increase over time and the intensity of POSTN expression decreases over time. Within bone calluses, we find the presence of MSI1 expression in mesenchymal stromal cells, osteoblasts, and osteocytes but not in hypertrophic chondrocytes. After 14 days, the expression of MSI1, POSTN, and RUNX2 was significantly correlated. Thus, we conclude that musashi-1 potentially serves in the osteogenic differentiation of mesenchymal stromal cells and bone healing. Therefore, further studies are needed to determine the possibility of musashi-1′s role as a clinical biomarker of bone healing and therapeutic agent for bone regeneration.

## 1. Introduction

Bone tissue has self-repair properties depending on mesenchymal stromal cells’ (MSCs’) recruitment and differentiation, angiogenesis, formation of a bone fracture callus, and endochondral and intramembranous bone ossification. MSCs can be found in the bone marrow, endosteum, and periosteum. Thus, during bone repair, these are the main sources of cells for the healing process [1]. That is the reason why MSCs have been proposed for the treatment of several bone diseases.

The Musashi family is a group of two RNA-binding proteins with two ribonucleoprotein-type RNA-binding domains (RBDs) [2]. The function of Musashi-1 (MSI1, gene Msi1) is exerted by indirectly activating Wnt through a slower degradation of ß-catenin [3]. It also blocks p21^WAF−1^ [4], an inhibitor of the Wnt pathway. On the other hand, MSI1 inhibits *numb*, whose main function is to block the actions of Notch on maintaining the self-renewal capabilities of stem cells [5]. Taken together, this evidence shows that MSI1 acts as translational regulator of target mRNAs to maintain the stemness and self-renewal capabilities of undifferentiated cells [6].

A role of MSI1 has been previously suggested in bone repair by demonstrating an increased detection in articular joints under repair after induction of arthritis [7]. This can be explained by the interaction of MSI1 with the Notch and Wnt/ß-catenin pathways. In the downstream of the mentioned pathways, runt-related transcription factor 2 (RUNX2, gene Runx2) appears as the main focal point for signaling integration [8]. RUNX2 is necessary for the maturation of chondrocytes [9]. When expressed in conjunction with osterix, the differentiation is directed towards osteogenic tissues [10].

A cross-talk between some of these pathways involves members of the transforming growth factor-beta (TGFß) family such as the bone morphogenetic proteins (BMPs) [11]. TGFß-1, in particular, plays a fundamental role in inducing the expression of periostin (POSTN, gene Postn), a matricellular molecule highly expressed in mechanically demanding tissues such as dental pulp [12], periodontal ligament [13,14], periosteum, and cardiac valves [15]. POSTN has been shown to promote cell mobility, survival, and proliferation in a number of cell types [16,17,18], to act as a key regulator of bone formation via its interactions with BMP and Wnt pathways [19] and to increase collagen cross-linking and extracellular matrix stability [20], necessary for supporting later mineralization in bone and dental tissues. In fact, POSTN deficiency increases bone damage and alters the reparative process [21]. Not surprisingly, serum levels of POSTN have been proposed as markers of bone metabolism [22].

Thus, the aim of this study was to evaluate the expression of Musashi-1 in the context of bone healing in a preclinical model of long bone fracture and correlate it with the expression of other osteogenic markers, including RUNX2 and POSTN. Results will be compared with those of non-fractured controls with the hypothesis that the expression is higher in the bone callus.

## 2. Results

In the present study, we evaluate the number and distribution of MSI1 positive cells in different tissue compartments of the bone calluses after inducing femoral diaphysis fractures without osteosynthesis. We then analyze the relation between MSI1, RUNX2, and POSTN-positive cells within different tissue compartments and their overall mRNA expression.

### 2.1. Histomorphometric Study

**Figure 1:**
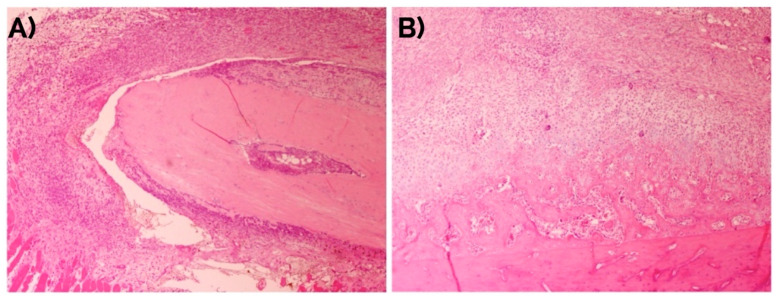
Evolution of the bone fracture callus at days (**A**) 4 and (**B**) 7. Hematoxylin-eosin. Original magnification: 4×.

**Figure 2:**
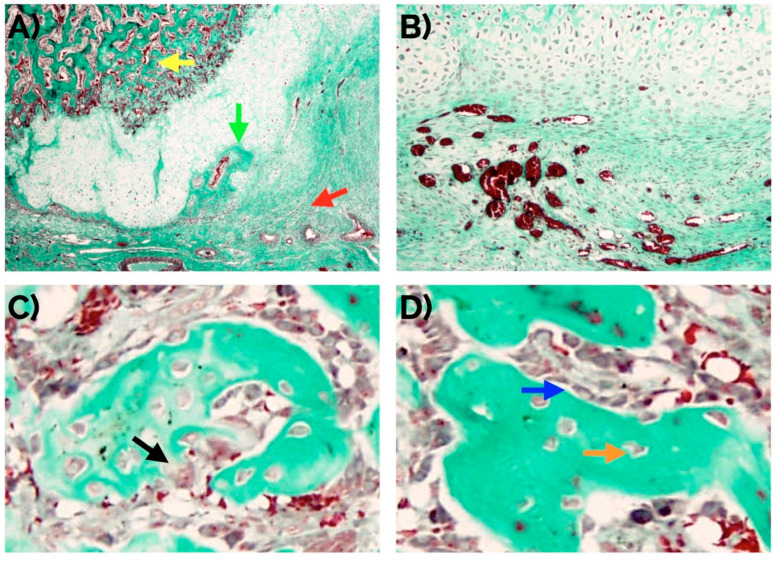
(**A**) Histomorphological confirmation after 14 days of an effective endochondral and intramembranous repair around a bone fracture callus involving fibrocartilage (red arrow), cartilage (green arrow), and newly formed bone trabecula (yellow arrow). An adequate number of new vessels can be observed (**B**) as well as normal distribution of osteoclasts (arrow in (**C**)), and osteocytes (orange arrow in (**D**)), and osteoblasts (blue arrow in (**D**)). Masson’s trichrome staining. Original magnification: 4× (**A**), 10× (**B**) and 40× (**C**,**D**).

The fracture site after four days already showed the presence of fusiform mesenchymal cells in a moderate proportion (72% of the incipient callus area). These cells were located between the striated muscle cells with the presence of mild interstitial edema without hematoma. At day seven, the proportion of mesenchymal cells (49%) was reduced and areas of mature and immature hyaline cartilage predominated (39%) with formation of bone trabeculae (12% of the callus area) (Figure 1). At day 14 (Figure 2), the presence of mature hyaline cartilage (50%) and formation of bone trabeculae generated through enchondral ossification (41.7%) was more evident. On average, newly formed bone trabecula contained 850.9(262.5) bone-forming cells in the interface between mineralized and non-mineralized areas of the bone (i.e., osteoblasts), 451.0(60.1) cells inside lacunae in the mineralized areas of the bone (osteocytes), and 31.3(18.4) multinucleated cells adjacent to mineralized areas of the bone (osteoclasts) per mm^2^.

### 2.2. Immunohistochemical Analyses

#### 2.2.1. Musashi-1

**Figure 3:**
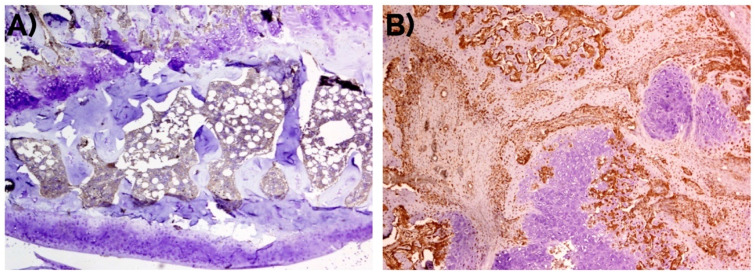
Negative detection of musashi-1 at day 14 in (**A**) unfractured controls in comparison with (**B**) bone fracture callus, as also represented in Figure 1. Peroxidase-conjugated micropolymer detection. Original magnification: 10×.

In unfractured controls, after 14 days, cells in the fibroconnective tissue showed weak to none MSI1 staining, in contrast with those in the bone fracture callus (Figure 3). MSI1 was not detected in mature adipocytes (not shown).

**Figure 4:**
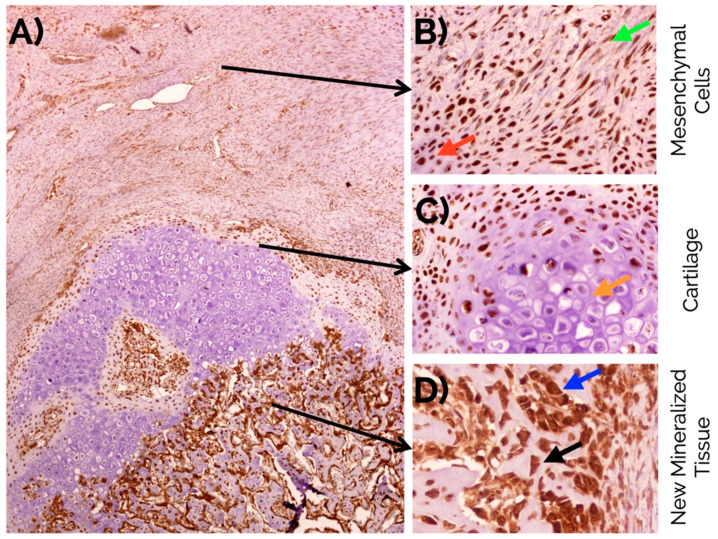
(**A**) Overview of musashi-1 detection in a bone fracture callus (in brown) and detail observations in (**B**) fusiform mesenchymal cells (green arrow), and immature chondrocytes (red arrow) in the fibrocartilage, (**C**) negative detection in mature hypertrophic chondrocytes (orange arrow), and (**D**) intense detection in the nucleus and cytoplasm of osteoblasts (blue arrow), and osteocytes (black arrow) in the newly formed bone. Peroxidase-conjugated micropolymer detection in samples from the 14-day time point. Original magnification: 10× (**A**), 20× (**B**,**C**) and 40× (**D**).

An intense nuclear expression of MSI1 was observed in fusiform mesenchymal cells and immature chondrocytes in the fibrocartilage. In osteoblasts, MSI1 was detected in the nucleus and the cytoplasm. MSI1 was not detected in mature hypertrophic chondrocytes (Figure 4).

**Figure 5:**
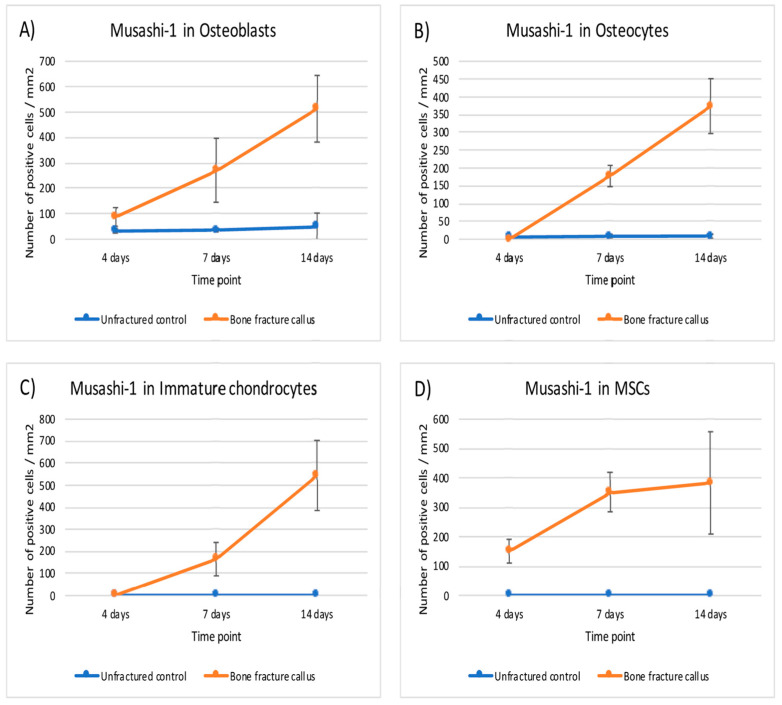
Graphical representation of the expression of musashi-1 in bone fracture callus (orange line) and unfractured control areas (blue line) over time in (**A**) osteoblasts, (**B**) osteocytes, (**C**) immature chondrocytes and (**D**) MSCs (mesenchymal stromal cells).

This expression of MSI1 increased over time from day 4 to day 14. The difference in number of positive MSI1 osteoblasts, osteocytes, immature chondrocytes, and mesenchymal cells per mm^2^ in the bone fracture callus compared to unfractured controls was statistically significant (*p* < 0.05, Mann–Whitney U test) at almost all time points (Table 1 and Figure 5).

A positive and statistically significant correlation was found at day 14 between the expression of MSI1 in MSCs and osteogenic cells (rho = 0.841, *p* < 0.001, Spearman test) (Table 2).

#### 2.2.2. RUNX2

Unfractured control bone did not show staining for RUNX2 in the cortical or trabecular bone. In the periosteum, a weak expression was visible in the closest areas to the cortical while the rest was negative. Similarly, a very weak staining was detected in the hematopoietic and lymphoid cells of the bone marrow.

**Figure 6:**
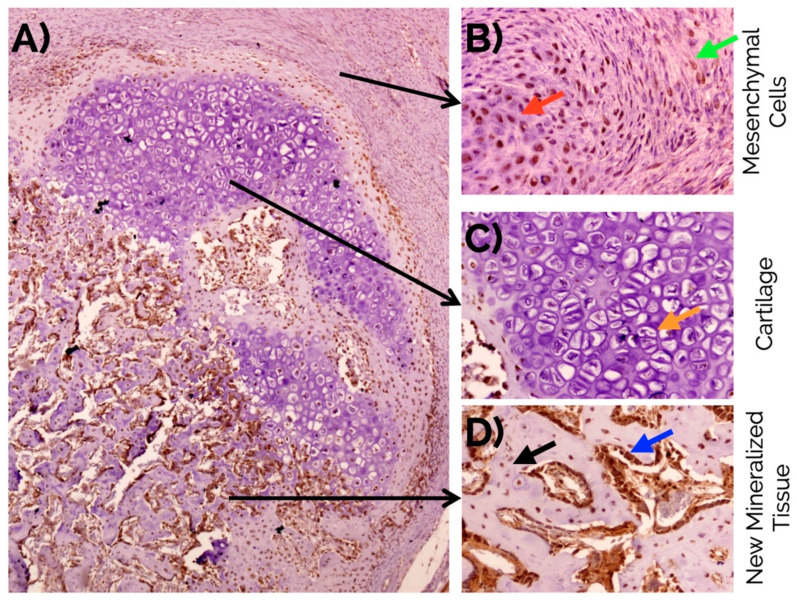
(**A**) Overview of RUNX2 detection in a bone fracture callus (in brown) and detail observations in (**B**) fusiform mesenchymal cells (green arrow), and immature chondrocytes (red arrow) in the fibrocartilage, (**C**) negative detection in mature hypertrophic chondrocytes (orange arrow), and (**D**) intense detection in the nucleus and cytoplasm of osteoblasts (blue arrow), and osteocytes (black arrow) in the newly formed bone. Peroxidase-conjugated micropolymer detection in samples from the 14-day time point. Original magnification: 10× (**A**) and 20× (**B**–**D**).

In the callus, an intense nuclear expression of RUNX2 was detected after 14 days similarly to the detection of MSI1 in MSCs and immature chondrocytes; similarly, RUNX2 was not detected in mature hypertrophic chondrocytes (Figure 6).

**Figure 7:**
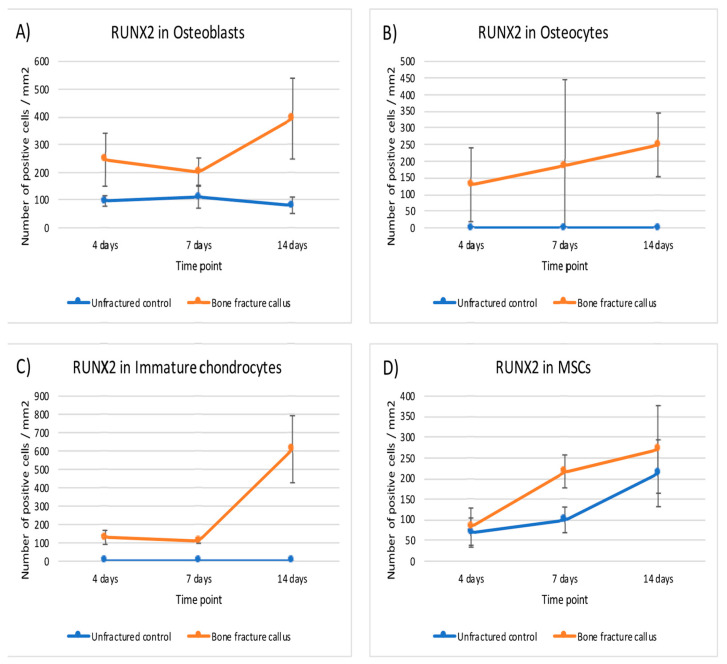
Graphical representation of the expression of RUNX2 in bone fracture callus (orange line) and unfractured control areas (blue line) over time in (**A**) osteoblasts, (**B**) osteocytes, (**C**) immature chondrocytes and (**D**) MSCs (mesenchymal stromal cells).

This expression did not change notably over time except in MSC and immature chondrocytes. The expression of RUNX2 was statistically higher in osteoblasts, osteocytes and immature chondrocytes of the fracture callus than in unfractured controls at all time points. Although the differences in mesenchymal cells were significant at seven days, it was rather marginal to none at day 4 and 14 (Table 3 and Figure 7).

**Figure 8:**
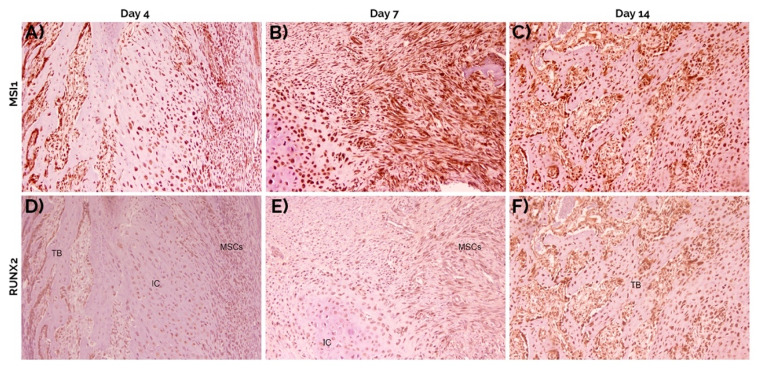
Staining of consecutive sections at 4 (**A**,**D**), 7 (**B**,**E**) and 14 days (**C**,**F**) for musashi-1 (**A**–**C**) and RUNX2 (**D**–**F**). Both markers increased over time. In general, the same cell types expressed both musashi-1 and RUNX2. Peroxidase-conjugated micropolymer detection. TB: trabecular bone; IC: immature chondrocytes; MSCs: mesenchymal stromal cells. Original magnification: 10×.

Although low, there was a positive significant correlation at day 14 between the expression of RUNX2 in MSCs and osteogenic cells (rho = 0.204, *p* ˂ 0.001, Spearman test). The expression of RUNX2 and MSI1 in osteogenic cells was strongly and significantly correlated (rho = 0.852, *p* ˂ 0.001, Spearman test) (Table 2 and Figure 8).

#### 2.2.3. Periostin

**Figure 9:**
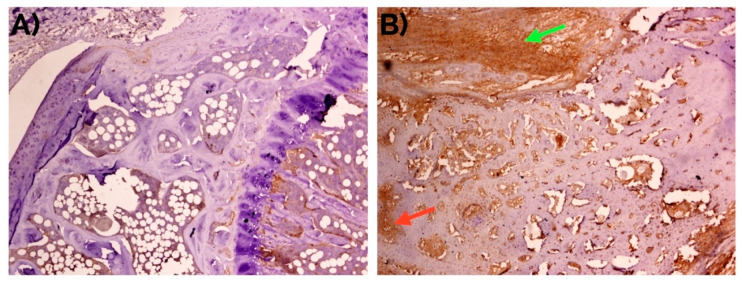
Weak to negative detection of periostin at day 14 in (**A**) unfractured controls in comparison with (**B**) bone fracture callus, where it can be observed mainly in the fibrocartilage (green arrow) and in the intertrabecular interstitium (red arrow). Peroxidase-conjugated micropolymer detection. Original magnification: 10×.

The immunohistochemical detection of POSTN was mainly interstitial. Over time, the intensity of POSTN expression decreased from 2.8 (0.4) after 4 days, to 2.6 (0.5) at day 7, and 1.8 (0.4) at day 14. After 14 days, POSTN was intensely detected in the fibrocartilage, specifically in the areas occupied by MSCs, and focally in the cytoplasm of mature hypertrophic chondrocytes (Figure 9). In the forming bone, there was an intense positivity in the intertrabecular interstitium. The cortical and trabecular bone as well as the cartilage tissue did not express POSTN. In the periosteum, the detection was weak/moderate.

The expression of POSTN was higher in the bone fracture callus than in unfractured control areas (*p* < 0.001; Chi-square test). The comparisons between the expression in fibrocartilage (*p* < 0.001; Chi-square test) and periosteum (*p* = 0.013; Chi-square test) were also statistically significant.

A positive correlation was found between the expression of POSTN and MSI1 in osteogenic cells in the fibrocartilage (rho = 0.881, *p* ˂ 0.001, Spearman test) but negative in the periosteum (rho = −0.820, *p* ˂ 0.001, Spearman test). Similar correlations were found with the expression of RUNX2 in the same cells and locations (rho = 0.933 and rho = −0.782, respectively; *p* ˂ 0.001, Spearman test) (Table 3).

### 2.3. mRNA Evaluation

**Figure 10:**
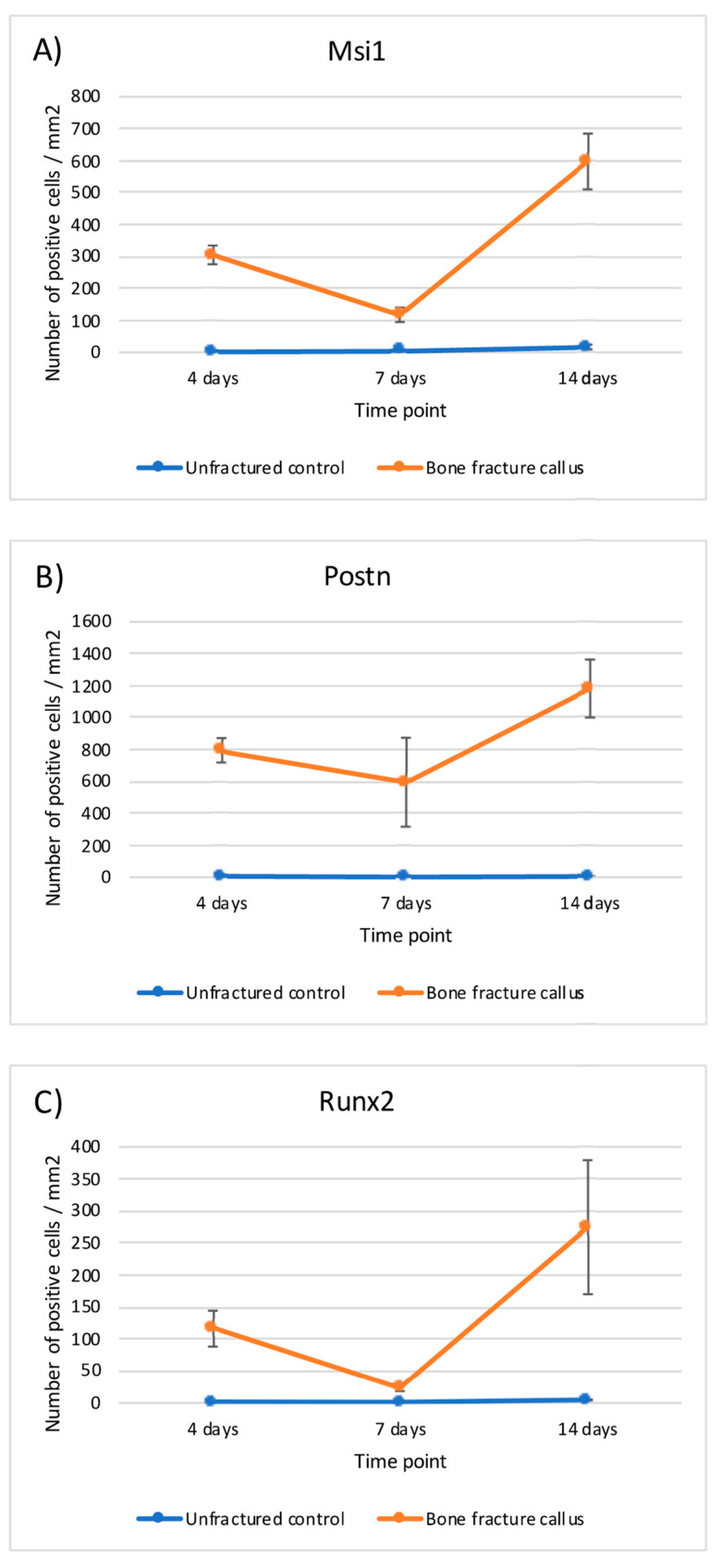
Graphical representation of the relative mRNA expression over time in bone fracture callus (orange line) and unfractured control areas (blue line) of (**A**) Msi1, (**B**) Postn and (**C**) Runx2.

The evaluation of the expression of Msi1, Postn, and Runx2 mRNA was in accordance with the protein detection by immunohistochemical analyses. In summary, higher expression of Msi1, Postn, and Runx2 was detected in the bone fracture calluses compared to unfractured controls at all time points. The differences between fractured and unfractured bone were statistically significant at day 4 and 14 (ANOVA model with Sidak’s multiple comparison test) (Table 4 and Figure 10).

## 3. Discussion

In the current study, the detection of RUNX2, POSTN, and MSI1 proteins and thir mRNA expression during bone repair is reported for the first time in the literature, as far as we know. This correlation highlights a potential role of Musashi-1 in the osteogenesis process during bone repair and regeneration. We have to keep in mind that this is a descriptive study. Thus, our findings need further confirmation. Yet, those findings are novel and interesting and set the basis for future analyses.

The animal model we selected presents a number of advantages. First, it is known that bone remodeling in rodents is similar to that in humans. Second, it is a well-characterized model. The femur also presents some advantages compared to other models in tibiae, being the most important at regular and comparable macroscopic diameter across animals. We did not perform osteosynthesis on the bone fractures because osteosynthesis complicates the procedure, increases the possibility of local infection, and gives less evidence of a histopathological response in terms of bone callus formation. Subchondral areas of non-fractured bone were used as control because, in bone fracture models, osteosynthesis is achieved primarily through endochondral ossification, as opposed to direct ossification (“*per priman* consolidation”) that occurs when true stabilization is achieved. Furthermore, the use of subchrondral areas allowed us to observe articular cartilage and the growth plate. Thus, in our opinion, the use of this control is appropriate. However, this methodology is temporarily uncomfortable for the animals so the veterinarian was especially careful with their welfare.

Evaluating the samples at different time points up to 14 days allowed an overview of the process. After 14 days, a reduction of the inflammatory infiltrate and granulation tissue and a more mature osseous tissue was observed, as well as a good number of vessels per mm^2^, a key factor for bone fracture healing [23]. This observation timeframe also allowed the endochondral and intramembranous formation of new trabecular bone with low remodeling activity, as is shown by the number of osteogenic cells. The use of a model of bone fracture without immobilization was chosen to observe the natural healing process and the implication of mechanical activity.

The expression of musashi-1 in osteoarticular tissues was reported for the first time in a model of induced arthritis. An intense nuclear expression of musashi-1 was demonstrated in the presence of reparative processes in response to arthritic lesions that was absent in healthy joints [7]. In order to further evaluate these findings, an in vitro study was conducted to analyze the expression of musashi-1, periostin, and RUNX2 in mesenchymal stromal cells obtained from different origins within the oral cavity and induced to differentiate into osteogenic cells. A clear correlation between the expression of musashi-1 and RUNX2 was found [24], which is similar to the results from the current study. Clinically, a preliminary study demonstrated that musashi-1 was mainly detectable in regenerated bone, particularly in osteoprogenitor mesenchymal cells of biopsies from previously grafted bone. In contrast, musashi-1 was almost undetectable or with very low levels in native non-regenerated bone within the same biopsy [25]. Furthermore, a higher expression of musashi-1 has been observed in association with bone grafting biomaterials that also induce higher presence of osteoblasts, osteocytes, osteoclasts, and osteoid lines in human bone biopsies [26,27,28,29]. The results from the current study localize the intense expression of musashi-1 in cells in the fibrocartilage compartment of the bone fracture callus (mesenchymal cells and immature chondrocytes). Thus, taking all this evidence together, the potential role of musashi-1 in bone repair can be proposed to be linked to the activity of mesenchymal stromal cells with differentiation potential, particularly those in the osteolineage path. The clinical relevance of these findings ranges from the use of musashi-1 as a marker of bone healing to a more complex application as an inductor of bone healing. This latter potential application is still in a very early stage and needs further investigation.

The current study also tries to put the expression of musashi-1 in the context of different bone healing times and tissue compartments. Musashi-1 has been related with the self-renewal capacities of MSCs and other cell types with high proliferative potential, such as those from osteosarcoma [30]. The actions of musashi-1 take place both during development and in the adult tissues [31]. In osteogenic progenitors, these actions are oriented to inhibit three main targets: *numb*, *p21^WAF−^*^1^, and the accumulation of ß-catenin, which block the activities of the Wnt and Notch pathways [32]. The accumulation of ß-catenin has also been suggested as one of the inductors of musashi-1 in MSCs during the activation of a reparative process [33]. Thus, we could argue that the mechanisms that take place in bone healing might be similar to those previously described in other tissues.

Two of the Wnt/ß-catenin and Notch downstream targets are BMP2 and RUNX2, probably the better described regulators of osteoblastic differentiation [8]. In this sense, the expression of RUNX2 that we detected, particularly in the bone fracture callus [34], can be explained by the previously described implication of RUNX2 in the transition from mature chondrocytes to hypertrophic chondrocytes [35], and its induction of endochondral and intramembranous ossification [36], in both cases dependent on the Wnt pathway. In these differentiation pathways, osterix is also important in the cell-fate decision process that will determine if mesenchymal stromal cells will become chondrocytes and osteoblasts or other cell type [37]. Thus, our findings regarding the mRNA expression of Runx2 in the context of bone fracture healing are within the previously described mechanisms. This, and the positive correlation of the immunohistochemical expression of both RUNX2 and musashi-1, in turn, offer an indirect confirmation of the importance of Musashi-1 the process as we have described.

During bone healing, even before the differentiation of MSCs is induced, a competent extracellular matrix is needed. Periostin is induced as a consequence of the activation of latent extracellular TGF-ß1. The induced periostin interacts with tenascin-C, fibronectin, and BMP1 to activate the lysyl-oxidase enzyme. This interaction increases collagen fibrillogenesis and cross-linking [20]. This is fundamental for bone strength. In addition, periostin may directly reduce the expression of sclerostin [38]. In fact, periostin upregulation has been observed in conjunction with an in vitro overexpression of RUNX2 [39]. Taken together, this evidence further supports our findings regarding the correlations between the expression of RUNX2, musashi-1, and periostin. Other evidence from similar experiments as the one performed in the current study also confirms the upregulation of Postn mRNA in undifferentiated mesenchymal cells and pre-osteoblasts in the callus compared to unfractured bone [40,41].

**Figure 11:**
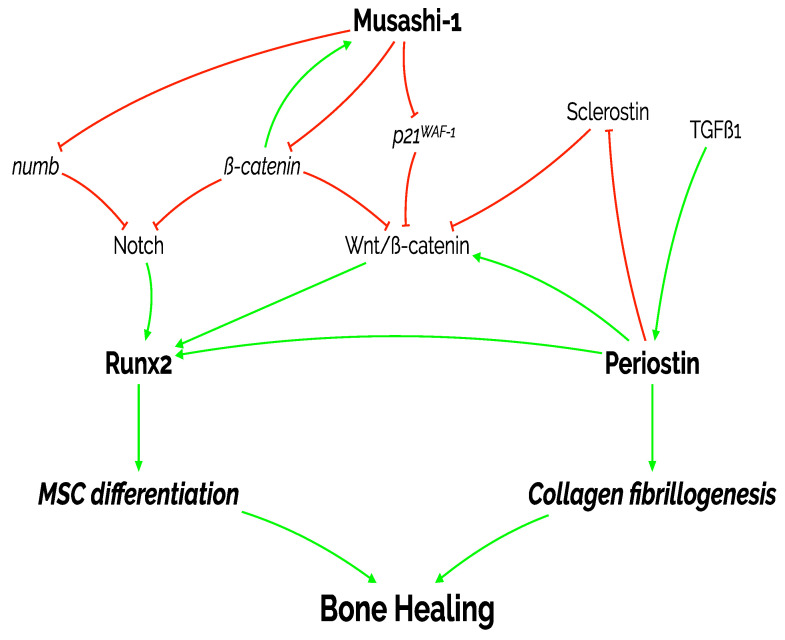
Diagram representing the proposed actions of the three molecules under study on MSC differentiation, collagen fibrillogenesis and, ultimately, bone healing. As shown in this manuscript, there is a positive and statistically significant correlation between the expression of the three markers: periostin, RUNX2, and musashi-1. MSC: mesenchymal stromal cell; p21^WAF−1^: cyclin-dependent kinase inhibitor 1; TGFß1: transforming growth factor beta 1.

Our study also presents some limitations, the most important being that we have not tested any mechanistic association between the molecules under analysis. Our findings regarding the correlation between musashi-1, RUNX2, and periostin in bone healing over time and in different areas of the bone callus suggests their importance in this context. However, a confirmation of such interplay would come from more sophisticated studies using knockout in vivo models and blocking of molecular pathways in vitro. Regardless, the information presented here is worth as no previous information about musashi-1 in the context of over time in vivo fracture bone healing has been published. Thus, the information presented in this study and the context explained above allow us to propose a very preliminary molecular model, as represented in Figure 11. Future studies will need to confirm if the proposed interconnection truly occurs.

## 4. Materials and Methods

### 4.1. Preclinical Model

A total of 46 samples from adult (8–9 week old) male Wistar rats (unfractured control: *n* = 23; fracture: *n* = 23), weighing 300–350 g, were used in this study. The animals were housed in stable conditions of light with ad libitum access to water and food. All experiments were performed after the approval of the Committee on Animal Research of the University of Granada (CEEA 2014/357) and under the European Union and Spanish regulations for ethics in animal research (EU Directive 63/2010 and Spanish RD 53/2013) and reported following the ARRIVE guidelines.

### 4.2. Surgical Procedure

A model of bone fracture without osteosynthesis nor external immobilization was used to observe endochondral and intramembranous repair [42]. Bone fractures were performed on the femoral diaphysis of the right hind limbs. Briefly, the rats were anesthetized by inhalation of ether followed by intraperitoneal injection of a solution containing ketamine (1 mL), diazepam (0.8 mL), and atropine (0.2 mL) at a dosage of 3 mL/kg. A blunt guillotine was dropped on the hind limb to produce the bone fracture. Post-operative analgesia was achieved by administration of 200 mg/kg of metamizole in the drinking water and 50 mg/kg of ibuprofen subcutaneously every 12 h during 3 days.

The rats were euthanized by CO_2_ overdose after 4, 7, and 14 days. Seven rats were euthanized after each time period, all extra rats were euthanized after 14 days as well. Secondary method of sacrifice consisted on the induction of pneumothorax.

### 4.3. Histomorphometric Study

The collected whole hind limbs were immediately fixed in a 10% formalin buffer after removal of major soft tissues. After 48 h at 4 °C, samples were decalcified in Decalcifier I^®^ (Surgipath Europe Ltd., Peterborough, United Kingdom) for 1 week at 37 °C and embedded in paraffin. Sections of 4 µm thickness were obtained along the major axis of the bone and treated for hematoxylin-eosin, PAS, and Masson’s trichrome stainings.

**Figure 12:**
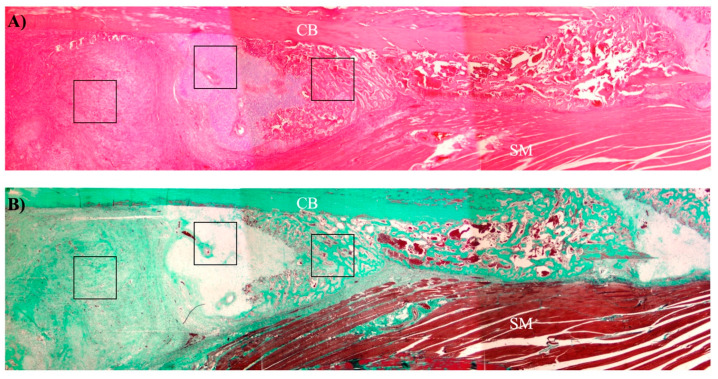
Representative low magnification image of a bone callus showing the approximate areas of each tissue compartment. (**A**) Hematoxylin-eosin staining. (**B**) Masson’s trichrome staining. CB: cortical bone; SM: skeletal muscle; black squares: different tissue compartments of bone callus (from left to right: connective tissue, cartilage, and new bone tissue).

Histomorphometric analysis was performed semiautomatically with the ImageJ software on Masson’s stained sections by evaluating a total of 10 images captured randomly from 3 sections of each fracture callus, adjacent tissues, and unfractured control bones. Images were obtained with a BX51 microscope equipped with a DP70 digital camera (Olympus Optical Company, Tokyo, Japan). Areas of connective tissue, cartilage, and new bone tissue (identified morphologically as represented in Figure 12) were measured as well as cells and vessels in each compartment.

### 4.4. Immunohistochemical Analyses

Paraffin-embedded sections were dewaxed, hydrated, and heat-treated in 1 mM EDTA (pH 8) for antigenic unmasking in an antigen retrieval PT module (Thermo Fisher Scientific Inc., Waltham, MA, USA) at 95 °C for 20 min. A total of three sections were incubated with polyclonal antibodies against MSI1 (Sigma-Aldrich, Barcelona, Spain; 1:100 during 16 h at 4 °C), POSTN (Santa Cruz Biotechnology Inc., CA, USA; 1:50 during 1 h at room temperature), and RUNX2 (Santa Cruz Biotechnology; 1:80 during 10 min. at room temperature). The micropolymer-peroxidase-based method (Ultravision Quanto, Thermo Fisher Scientific) was applied for the immunohistochemistry study with an automatic immunostainer (Autostainer 480S, Thermo Fisher Scientific), followed by development with diaminobenzidine. All antibodies and reagents were provided by Vitro-Master Diagnóstica S.A. (Granada, Spain). Appropriate positive and negative controls were run concurrently. Hematoxylin was used for nuclear counterstaining. To evaluate the co-localization of the expression of musashi-1 and RUNX2, consecutive sections were stained for each marker.

A millimeter scale in the eyepiece of a microscope (BH2, Olympus Optical Company) with a 40× objective was used to count the number of positive cells per mm^2^ in each tissue compartment. Cell type was identified morphologically. Results were expressed as numbers of positive cells/mm^2^/0.062 (correction value for 40× magnification). Extracellular expression of periostin was evaluated by a categorical scale (0 = none; 1 = weak; 2 = moderate; 3 = intense).

### 4.5. mRNA Evaluation

A group of animals were subjected to the same procedures with the purpose of obtaining tissue for mRNA analyses. After euthanasia, bone fracture calluses and unfractured control bone were collected without dissecting the periosteum. Tissues were immediately submerged in Trizol reagent (Trizol^TM^ Plus RNA Purification Kit, Invitrogen, Grand Island, NY, USA) and homogenized with a tissue blender before RNA from each individual sample was isolated. RT-PCR was performed using a thermal cycler (iCycler iQ^TM^, Bio-Rad Laboratories, S.A., Madrid, Spain) immediately after RNA isolation for a final volume of 30 μL of cDNA with 1 µg of RNA using the PrimeScript™ RT Master Mix (Takara Bio Europe, Saint-Germain-en-Laye, France). Table 5 shows the primers used in the quantitative PCR using iQ-SYBR Green Supermix (Bio-Rad). Each plate contained triplicates of the cDNA templates. The 2^−ddCt^ method was used to calculate gene expression levels relative to Gapdh. Normalization was made to the unfractured controls at day 4.

### 4.6. Statistical Analysis

Results are expressed as mean (standard deviation) for continuous variables with normal distribution and as frequencies for categorical variables. Statistical analyses were performed by Chi-square (for categorical variables), Mann–Whitney U (for continuous variables) and Spearman correlation tests with IBM SPSS 20.0 (Armonk, NY, USA). Differences among and between groups and time points in mRNA expression were statistically analyzed by a two-way ANOVA model with Sidak’s multiple comparison tests with Prism 7 (San Diego, CA, USA). In all cases, statistical significance was established at level of *p* below 0.05.

## 5. Conclusions

Bone healing, in the context of the current experiment and within the limitations already discussed, could be proposed to be a combination of the expression of periostin to stabilize the extracellular matrix and the release of RUNX2 as a result of the activity of previously well-described pathways that are also interconnected with the expression of musashi-1. The increased expression over time of musashi-1 in cells compounding the bone fracture callus, together with RUNX2 and periostin, suggests a potential role as putative regulator in the bone healing process. This observation and its significance remain to be solved with more detailed, mechanistic studies.

## Figures and Tables

**Table 1:** Comparison between the expression of Musashi-1 in bone fracture callus and unfractured control areas.

Expression of Musashi-1 (Positive Cells/mm^2^)	Unfractured Control	Bone Fracture Callus	Mann–Whitney U Test (*p*-Values)
Osteoblasts (4, 7, and 14 days)	32.0 (8.8)	87.2 (37.2)	0.024
	35.2 (7.4)	270.8 (126.4)	0.008
	48.3 (53.4)	512.9 (131.7)	0.001
Osteocytes (4, 7, and 14 days)	6.0 (2.9)	0.0 (0.0)	0.007
	8.4 (5.0)	177.4 (30.3)	0.012
	8.0 (5.7)	373.8 (77.4)	0.001
Immature chondrocytes (4, 7, and 14 days)	0.0 (0.0)	0.0 (0.0)	>0.999
	0.0 (0.0)	164.6 (75.0)	0.008
	0.0 (0.0)	544.3 (158.3)	0.001
Mesenchymal cells (4, 7, and 14 days)	0.0 (0.0)	150.6 (39.8)	0.008
	0.0 (0.0)	351.6 (66.8)	0.007
	0.0 (0.0)	383.0 (173.5)	0.001

Values are expressed as mean (SD).

**Table 2:** Correlations between the expression of the different markers under study at day 14.

Marker	Osteogenic Cells			Mesenchymal Cells	Rho (*p* < 0.01, Spearman)
Musashi-1	X	X		X	0.841
RUNX2	X		X	X	0.204
Periostin		X	X		
Rho (*p* < 0.01, Spearman)	0.852	0.881	0.933		

**Table 3:** Comparison between the expression of RUNX2 in bone fracture callus and unfractured control areas.

Expression of RUNX2 (Positive Cells/mm^2^)	Unfractured Control	Bone Fracture Callus	Mann–Whitney U Test (*p*-Values)
Osteoblasts (4, 7, and 14 days)	96.1 (19.0)	245.2 (95.9)	0.008
	111.4 (41.3)	200.6 (51.3)	0.016
	80.6 (29.6)	393.3 (146.1)	0.002
Osteocytes (4, 7, and 14 days)	0.0 (0.0)	129.2 (111.3)	0.008
	0.0 (0.0)	187.0 (258.2)	0.007
	0.0 (0.0)	249.1 (95.9)	0.001
Immature chondrocytes (4, 7, and 14 days)	0.0 (0.0)	129.0 (37.5)	0.007
	0.0 (0.0)	109.8 (13.4)	0.007
	0.0 (0.0)	610.2 (182.1)	0.001
Mesenchymal cells (4, 7, and 14 days)	69.0 (35.0)	83.4 (44.7)	0.548
	99.8 (30.7)	217.0 (40.0)	0.008
	212.9 (80.7)	270.6 (105.9)	0.190

Values are expressed as mean (SD).

**Table 4:** Relative mRNA expression of Msi1, Postn, and Runx2 in the different areas under study. Note the significant differences between detection of Msi1, Postn, and Runx2 in fracture and unfractured controls, particularly at day 4 and 14. *: *p* values for the comparisons of unfractured control vs. callus (Sidak’s multiple comparisons post-hoc test).

mRNA	Unfractured Control	Bone Fracture Callus	Adjusted Sidak Test *
Msi1 (4, 7, and 14 days)	1.00 (0.38)	304.16 (29.77)	<0.001
	4.75 (0.47)	116.09 (22.61)	0.086
	15.24 (7.19)	596.62 (87.96)	<0.001
Postn (4, 7, and 14 days)	1.00 (0.24)	793.40 (77.65)	0.004
	0.81 (0.20)	593.63 (279.84)	0.016
	4.85 (0.24)	1179.38 (181.43)	<0.001
Runx2 (4, 7, and 14 days)	1.00 (0.19)	115.98 (28.14)	0.117
	0.52 (0.13)	22.99 (5.06)	0.929
	4.12 (0.60)	274.22 (104.48)	0.003

Values are expressed as mean (SD).

**Table 5:** Primer sequences for the mRNA evaluation of the specified genes.

Gene	Forward	Reverse
Gapdh	5-GTGAAGCTCATTTCCTGGTATGAC-3	5-GCCTCTCTCTTGCTCTCAGTATC-3
Msi1	5-ACGTTCGAGAGTGAGGACATC-3	5-CCTCCTTTGGCTGAGCTTTC-3
Postn	5-AACCCGGAGTCACCAACATC-3	5-GCGTCTCATTGACTCCTTTCAC-3
Runx2	5-AGTGATTTAGGGCGCATTCC-3	5-TCTGCCTGGGATCTGTAATCTG-3

## Data Availability

The data that support the findings of this study are available from the corresponding author upon reasonable request.
